# Examining the Methods Adolescents Use in Nonsuicidal Self‐Injury: A Multi‐Wave Latent Profile Analysis

**DOI:** 10.1002/jad.12516

**Published:** 2025-05-28

**Authors:** Lauree C. Tilton‐Weaver, Sheila K. Marshall, Ylva Svensson

**Affiliations:** ^1^ Örebro University Örebro Sweden; ^2^ University of British Columbia Vancouver British Columbia Canada; ^3^ University West Trollhättan Sweden

## Abstract

**Introduction:**

Nonsuicidal self‐injury (NSSI) among adolescents is a health concern. Little is known about the *patterns* of methods adolescents use, in terms of how many and how often different methods are used.

**Methods:**

We used three annual waves of data from 630 Swedish adolescents (T1: age 12–18 years; 56% girls), who reported NSSI use at least once. Latent profile analysis was used to examine profile differences, with supplementary analyses focused on differences and change predicted by gender, internalizing, emotion dysregulation, interpersonal stressors, and severity of NSSI.

**Results:**

Three profiles consistently emerged over time: one very *low* in NSSI, another with higher frequencies of *cutting/scraping* skin, and one reporting *multiple methods* of NSSI, ranging from moderate (T1) to high (T3) frequency. Profile subgroups differed: *low* subgroups consisted of the fewest girls and reported the lowest levels of intra‐ and interpersonal issues. Additionally, subgroups differed in severity of NSSI, suggesting damage to the skin may not be the only reason medical attention is needed. Significant change in subgroup membership was also observed.

**Conclusions:**

Although most adolescents engaged in NSSI at very low rates, many used multiple forms, differing in both frequency and versatility. Few differences were found between subgroups characterized by higher frequencies, suggesting that it might be possible to identify adolescents most in need of treatment by attending to the methods most frequently used. Results also suggested that measuring the severity of each method may yield more accurate information than a priori groupings.

## 1 Introduction

Nonsuicidal self‐injury (NSSI) is a health concern during adolescence as it is associated with other psychological problems, including future risk of suicidality (Brausch and Woods 2019; Guan et al. 2012). Although overall rates of NSSI, its functions, and correlates have been systematically studied, less is known about why adolescents use some methods of self‐injury and not others. Research focusing on each method of NSSI separately, as opposed to aggregated measures, suggests there is a good deal of variation in the ways older adolescents (e.g., Somer et al. 2015) and young adults (e.g., Klonsky and Olino 2008) engage in NSSI. This variation suggests that adolescents and young adults do not uniformly use the same methods but differentially prefer some methods over others. Variation in the methods used are also likely in early adolescence and may be related to other aspects of NSSI use that could aid in early identification and treatment.

Early and middle adolescence are important periods for the development of NSSI. Although onset is most common between ages 14 and 17 years (Gandhi et al. 2018), it can occur earlier (Jacobson and Gould 2007; Klonsky et al. 2011; Nock 2009). Early adolescence is also a period in which emotional sensitivity and social challenges increase (Compas and Phares 1991; Heaven 2001). However, there is little information about what methods of NSSI are used during early to middle adolescence and how much they vary. We aim to address this gap.

### 1.1 Patterned‐Centered Approaches to Methods of NSSI

Research focused on NSSI has generally relied on variable‐centered approaches. That is, average frequency and changes on average frequency have been well‐explored (Hamza and Willoughby 2014), as have associations between NSSI and other variables. Such studies on adolescents suggest that frequency of NSSI is related to a diverse set of problematic experiences and characteristics, such as impulsivity, emotion dysregulation, depressive symptoms, substance abuse, disordered eating, self‐concept instability, self‐criticism, experiencing abusive treatment and stressful life events, and suicidality (Allen et al. 2019; Brausch and Boone 2015; Burke et al. 2015; Daly and Willoughby 2019; Lear and Pepper 2016; Titelius et al. 2018). Versatility has also been tied to a variety of variables, including NSSI severity, perceived stress, types of coping, self‐concept instability, and suicidality (Kiekens et al. 2015; Lear and Pepper 2016; Robinson et al. 2021; Victor et al. 2016). Although rare, explicit comparisons between NSSI frequency (how often NSSI is used) and versatility (the number of methods used) suggest that versatility is more strongly related to suicidality (Turner et al. 2013). These studies draw attention to the importance of examining both frequency and versatility.

Despite this body of evidence, the variable‐centered approach assumes that the sampled population is homogeneous in these associations. Such uniformity is unlikely, given that adolescents vary in the methods they use. Moreover, nearly all studies use aggregated scores as the NSSI variable, treating different methods as somewhat interchangeable (Bracken‐Minor et al. 2012). As a result, there is need for information on combinations of frequency and versatility in NSSI. In addition, identifying subgroups also allows researchers to examine what distinguishes one group from another, including potential antecedents and outcomes. As research suggests that mental health is related to the *patterns* of NSSI methods (Klonsky and Olino 2008), identifying subgroups who are similar in levels of frequency and versatility, and examining differences between subgroups on relevant precursors and outcomes could help guide theory and intervention.

Behavioral models of NSSI do not explicitly account for variation in frequency and versatility, but research examining groupings suggests that there may be important differences. For example, adolescents who are “experimenting” with NSSI may try a variety of methods only once or twice. Adolescents whose NSSI fits an experimental profile may desist on their own or benefit from intervention programs meant to address NSSI when it is relatively minor. Comparatively, other subgroups may need more immediate, direct, and intensive interventions. Such subgroups could include adolescents whose profiles indicate elevated use of NSSI methods that are considered severe or subgroups of adolescents whose profiles indicate high levels of frequency and versatility.

### 1.2 Previous Pattern‐Centered Research on NSSI Methods

Some research has focused on NSSI methods, using two approaches: either a priori groupings (e.g., Lloyd‐Richardson et al. 2007) or pattern‐centered analyses to identify homogeneous subgroups (e.g., Klonsky and Olino 2008). Many of these studies have found groups of older adolescents or young adults who engaged in very few or low frequencies of NSSI, with frequency aggregated across forms. Such subgroups have been described as “experimental” and/or “mild” for less severe NSSI (e.g., Bracken‐Minor et al. 2012; Case et al. 2020). In addition to these experimental youth, most studies find at least one grouping of young people who report repeated engagement in several methods (i.e., moderate to high versatility, e.g., Bjärehed et al. 2012; Bracken‐Minor et al. 2012). Another common finding is groups using one method, alone or more frequently than others (e.g., Bjärehed et al. 2012).

Although informative, these studies have limitations: (a) only three studies used adolescent samples, none that included early adolescents; (b) only one study was longitudinal, but short‐term (two annual waves, Bjärehed et al. 2012); (c) some used dichotomized reporting (use/no use, e.g., Reinhardt et al. 2022) removing information about the frequency of use; or (d) reported very small clusters (less than 1%, Bjärehed et al. 2012) that are unlikely to be replicated. Thus, research on variation in methods is rather static and missing information from early to middle adolescence. As NSSI tends to emerge and increase in frequency from early to middle adolescence (De Luca et al. 2023; Gandhi et al. 2018), it is important to document change in frequency and versatility of methods used, as there may be developmentally relevant change in NSSI across adolescents. Our first aim was to address this gap, using three longitudinal assessments covering this developmental period.

### 1.3 Differences Between Subgroups

Our second aim was to identify what differentiates subgroups. Drawing from Tatnell et al. framework (Tatnell et al. 2014; used by Wang et al. 2017), we examined *interpersonal experiences* that engender intensive negative emotions and *intrapersonal issues* or characteristics that amplify or fail to dampen emotional arousal. This framework is consistent with theoretical models emphasizing the role of interpersonal experiences and emotion dysregulation in NSSI (e.g., Nock 2010) and research suggesting that stressful events lead to NSSI via reducing individuals' ability to reduce emotional arousal (e.g., Ewing et al. 2019). This framework is also consistent with more general models of psychopathology, including those that emphasize gender differences (e.g., Rudolph 2009).

Generally, research shows that individuals experiencing interpersonal stressors engage in more frequent and versatile methods of NSSI. Such stressors include poor attachment (Cassels et al. 2019), abuse (Yates et al. 2008), difficulties with peers (victimization, Marshall et al. 2013); difficult relationships (Yurkowski et al. 2015), and romantic partners (Levesque et al. 2010). In studies that have examined interpersonal experiences in relation to methods of NSSI, the results are mixed. University students who reported using multiple methods also reported more abusive experiences, compared to those reporting one method (Whitlock et al. 2008). Others studying attachment, support, and belongingness have not found differences across groups (Singhal et al. 2021; Yates et al. 2008).

Comparatively, fewer studies have focused on how methods of NSSI are related to intrapersonal issues. Among the studies focused on intrapersonal issues, most have focused on psychological distress and internalizing symptoms (e.g., Somer et al. 2015). Both have been linked to greater NSSI frequency more generally (psychological distress: Baetens et al. 2014), depressive symptoms: Marshall et al. 2013). When subgroups are examined, both psychological distress and depressive symptoms are characteristic of subgroups exhibiting more versatility (Case et al. 2020; Klonsky and Olino 2008).

Others have focused on difficulties regulating negative emotions and ways individuals emotionally respond to negative experiences. Such studies show that NSSI is tied to difficulties regulating anger, including discomfort and difficulty controlling anger (Laye‐Gindhu and Schonert‐Reichl 2005), as well as anger suppression (Cipriano et al. 2020).

Given ties to symptoms of depression and anxiety, it is unsurprising that repetitive negative thinking, specifically rumination, is also related to NSSI (Coleman et al. 2022; Tilton‐Weaver et al. 2019). When these associations are studied in the context of subgroups, university students reporting more use of methods involving damaging the skin (scratch, piercing, banging, and bruising) also reported significantly more difficulties regulating emotions, compared to all other subgroups (i.e., those with elevated scratching and piercing, elevated burning, and an experimental group; Peterson et al. 2019).

Thus, both interpersonal and intrapersonal difficulties are related to engaging in NSSI. Yet, it is unclear how these issues and characteristics relate to the methods used during early and middle adolescence, as most studies have focused on older adolescents and young adults (university students).

In addition to interpersonal experiences and intrapersonal issues, we examined subgroup differences in terms of severity and gender. We examined subgroup differences in severity of NSSI because others have used a priori groupings, based on skin damage (e.g., Tatnell et al. 2014; Wang et al. 2017). If these groupings are warranted, subgroups of adolescents whose methods differ in bodily damage should differ in need for medical attention.

Regarding gender issues, some researchers examining NSSI have found no gender differences in methods used (Somer et al. 2015), while others have. In some studies, girls engaged in more cutting and scratching, whereas boys banged their heads, punched, or burned themselves (Barrocas et al. 2012; Sornberger et al. 2012). Thus, subgroups of adolescents who engage solely or primarily in cutting or methods of injuring the skin should consist of more girls than boys, whereas subgroups of adolescents engaging solely or primarily in banging, punching, or burning should include more boys.

In addition to the gender differences in methods used, girls tend to differ from boys in important correlates. Theoretical and empirical accounts suggest that girls are more distressed by interpersonal stressors, especially experiences with peers (Wagner and Compas 1990). Such distress is also tied to higher levels of internalizing symptoms and some forms of emotion dysregulation (e.g., rumination and worry). Here, too, are gender differences: compared to boys, girls experience more internalizing symptoms and associated repetitive negative thinking, particularly rumination (Bender et al. 2012; Nolen‐Hoeksema and Girgus 1994). By comparison, expressions of anger are generally considered less appropriate for girls than for boys (Chaplin and Aldao 2013). Thus, subgroups that are predominantly composed of girls (e.g., a subgroup high in versatility and frequency and subgroups using methods that damage skin) should report more interpersonal stressors and more difficulty with internalizing symptoms and repetitive negative thinking. Conversely, subgroups that are composed of more boys (e.g., a subgroup whose members engage primarily in burning, banging, or punching self) may report higher levels of dysregulated anger.

### 1.4 This Study

In this study, we used latent mixture modeling to examine what subgroups (latent profiles) would be found in two waves of longitudinal information from early and middle adolescents. We expected a profile of adolescents exhibiting low frequencies across multiple methods of NSSI (i.e., with moderate to high versatility), akin to the groups described as “experimental” in previous research (e.g., Bracken‐Minor et al. 2012; Somer et al. 2015). We also expected that this subgroup would be composed of more boys than girls or would have fewer girls than profiles with greater frequency and versatility.

We also expected a profile where engagement in NSSI included multiple methods, at higher frequencies, like those found by Somer et al. (2015). Because NSSI frequency increases over time for some adolescents (Tilton‐Weaver et al. 2023), we expected subgroups with higher frequencies at later waves, relative to earlier. We also expected the subgroups with higher frequency and versatility to be composed of more girls than boys.

Based on extant cross‐sectional research, we speculated that subgroups focused mainly on cutting or self‐battery might emerge (like Bjärehed et al. 2012). If so, we speculated that a subgroup with a high frequency of cutting would likely contain more girls than boys, whereas a subgroup focusing on self‐battery would contain more boys.

To explore differences between subgroups, we examined stressful experiences at home, with peers, and in school; and peer victimization (relational and physical) to tap interpersonal experiences. To represent intrapersonal issues, we examined internalizing symptoms and emotion regulation issues, including repetitive negative thinking and anger dysregulation.

Given the ties between NSSI and interpersonal stressors, we expected that subgroups exhibiting both high frequency and versatility would have the highest levels of stressors and victimization. Likewise, such subgroups should also report the highest internalizing symptoms and repetitive negative thinking. We speculated that subgroups whose members engaged primarily in cutting and scraping, especially at higher frequencies, would have the next highest levels of interpersonal stressors (including victimization), because they are expected to be composed of more girls than boys. A variation that we speculate may emerge is that a subgroup high in banging, punching, or burning (i.e., self‐battery) might have higher levels of anger dysregulation than the other subgroups, indicative of boys' masculine patterns of maladaptive coping. We expected the lowest levels of interpersonal stressors, internalizing symptoms, and all forms of emotion dysregulation to be reported by groups low in frequency.

Regarding severity, we expected subgroups with very low frequencies would report the lowest levels of severity (i.e., need for medical treatment). Comparatively, adolescents in subgroups including methods that cause skin damage (especially cutting, carving, and burning) should report higher levels of severity, with greater severity when more of these methods were also high in frequency. Such findings would provide evidence that a priori categorizations of severity based on skin damage are valid.

As there is no research on how subgroups change in early to middle adolescence, we put forward no firm hypotheses regarding transitions. Thus, analysis of change in subgroupings and predictors of change were exploratory.

## 2 Methods

### 2.1 Participants

Our sample came from a cross‐sequential project, conducted in 17 public secondary schools in three central Swedish municipalities. Using three annual assessments (covering 2 years), our target sample at T1 included a total of 3336 students in grades 7 and 8, ranging in age between 12 and 18 years (most were 13 or 14). Of those targeted, 2767 students whose parents passively consented participated (77% response rate). At T2, 3352 were targeted, of whom 2961 participated, including 2523 from T1 (9% attrition). At T3, 4038 were targeted, of whom 3022 participated, including 1983 from T1 (19% additional attrition).

As NSSI has a semi‐continuous distribution (with a large number of zeroes) and procedures for using such variables in latent classifications has not yet been developed, we restricted the analyses to participants reporting NSSI at least once in the three waves of assessment. This allowed us to examine the combinations of frequency and versatility. This resulted in a longitudinal sample of 630 adolescents, consisting of three cross‐sectional samples of adolescents reporting NSSI at that wave (*n*_*T*1_ = 346 of 2767, 22.8%; *n*_*T*2_ = 353 of 2961, 21.3%; *n*_*T*3_ = 316 of 3022, 20.8%). We eliminated the respondents who did not engage in NSSI at any time point.

This analytic sample consisted primarily of 13‐ and 14‐year‐olds at T1 (range 12–18, *M*_age_ = 13.59, SD = 0.67; 56% girls). The sample was mostly Swedish in origin (89.4% born in Sweden, 77.1% of mothers and 78.2% of fathers born in Sweden) with intact families (69.1% living with both parents; 64.9% married). Rough indicators of socioeconomic well‐being suggested that most adolescents lived in families with some disposable income (94.4% with at least one car, 85.9% taking annual family trips, and 92.5% with at least two computers at home). Demographic information is also provided for each subsample in Supporting Information: Table 1. As can be seen in that table, the demographics are relatively stable across waves and subsamples.

We estimated introduced bias by comparing those excluded from those included in the analytical sample. Binary logistic regressions showed that excluded adolescents were more likely to be older, report less NSSI, fewer internalizing symptoms, and less victimization (T3 only) (Nagelkerke *R* ^*2*^s were 0.10 for T1, 0.11 for T2, 0.15 for T3, all *p* < 0.001). These differences may have reduced variability, lowering power.

### 2.2 Missing Data

Within each wave, few data points were missing (1% at T1, 0.5% at T2 and 0.5% at T3). Little's Missing Completely at Random (MCAR) test, however, indicated that the data were not MCAR at T1 or T2 (T1: *χ* ^2^ = 717.93, *df* = 739, *p* = 0.02; T2: *χ* ^2^ = 411.22, *df* = 360, *p* = 0.03; T3: *χ* ^2^ = 329.24, *df* = 318, *p* = 0.32).

We tested for significant differences between cases missing data and those with full information, using binomial regressions including all of the demographic variables. At T1, we found that those missing data were more likely to be boys (75%) than girls (25%), when those with data were more equally distributed in terms of gender (41% boys and 59.4% girls) (*χ* ^2^[1] = 5.27, *p* = 0.02). No other differences were found at T1.

At T2, those missing data were more composed of more foreign‐born students (36.4%) than Swedish (63.6%), compared to those with data (91.4% Swedish, 8.6% foreign born) (*χ* ^2^[1] = 9.65, *p* = 0.002). They were also to have fathers born outside of Europe (45.5% Swedish, 9.1% born in another European country, and 45.5% born outside of Europe) compared to those with data (79.5% Swedish, 1.2% other Scandinavian, 6.0% other European, 13.4% born outside of Europe) (*χ* ^2^[3] = 9.48, *p* = 0.02). Finally, those missing data at T2 were more likely to in a living situation without both parents present (36.4% with both parents, 27.3% alternating between parents, 18.2% living with fathers only, 9.1% living with mothers only, and 2.4% with someone else) compared to those with complete data (66.4% with both parents, 12.4% alternating, 3.2% with fathers only, 15.6% with mothers only, and 9.1% with someone else) (*χ* ^2^[4] = 11.88, *p* = 0.02).

At T3, those missing data were more likely to have a mother born outside of Europe (40.0% born in Sweden, 60% born outside of Europe), compared to those cases with complete data (82.4% born in Sweden, 2.6% born in another Scandinavian country, 5.2% born in another European country, and 9.8% born outside of Europe) (*χ* ^2^[3] = 13.16, *p* = 0.004). Similar to those at T2, students missing data were more likely to have a father born outside of Europe as well (20.0% born in Sweden, 20.0% born in another Scandinavian country, and 60.0% born outside of Europe), compared to those with complete data (83.6% born in Sweden, 3.0% born in another Scandinavian country, 4.6% born in another European country, and 8.9% born outside of Europe) (*χ* ^2^[3] = 20.37, *p* < 0.001, 0.004). No other differences were found.

Overall, then, the patterns of missing data suggest that at T2 and T3, students with immigrant backgrounds may have been more reluctant to provide data than Swedish students. However, because NSSI was not significantly related to being born outside of Sweden, Scandinavia, and Europe, we conclude that the missing data did not unduly affect the profile analyses.

We treated the data as Missing at Random (MAR), using Robust Maximum Likelihood (MLR) to estimate our models, because our NSSI indicators were skewed. Further, we used Full Information Maximum Likelihood (FIML) to estimate the missing data, as its use provides more accurate estimates and standard errors than older missing‐data treatments (Enders 2010).

### 2.3 Procedure

Data were collected in classrooms during regular school hours by trained assistants. All parents were informed about the aim and duration of the study before each wave of data collection via regular mail, with opportunities to decline their adolescents' participation (via a postage‐paid card, phone call, or email). Overall, a total of 196 declined.

Before distributing questionnaires, students were told about the study aims and their rights (voluntary participation, with unimpeded withdrawal; data protection). Teachers were not present. Students were provided ample time to complete the questionnaire (including time for students to take breaks as needed). They received refreshments at mid‐point and each class received 300 SEK (≈30 US dollars) as an honorarium. The study was approved by the Regional Ethical Board in Uppsala, Sweden.

### 2.4 Measures

#### 2.4.1 NSSI

We assessed NSSI using a revised version of the Deliberate Self‐Harm Inventory adapted for adolescents (Gratz 2001; Lundh et al. 2007) and for assessing *non‐suicidal* self‐injury. Adolescents were asked how many times they had purposely used nine different methods of injury in the previous 6 months, without meaning to kill themselves. The methods included (1) cutting the wrist, arm or some other body part, (2) scratching themselves with a sharp object, on the arms or another part of the body, (3) burning themselves with a cigarette, lighter, or matches, (4) carved words, pictures, symbols, or something similar onto their skin, (5) scratched themselves so badly that it turned into a wound or started bleeding, (6) bitten themselves hard enough to tear a hole in their skin, (7) stuck sharp objects (like needles or something similar) into their skin (not counting tattoos, earrings, needles for medical purposes, or piercing), (8) hitting themselves or banging their heads against something so severely that it left a bruise, and (9) preventing wounds from healing. Possible responses ranged from 0 (*never*) to 6 (*more than 5 times*).

For *severity*, we used a single item tapping how the times that NSSI needed a hospital visit or medical treatment, with the same possible range.

#### 2.4.2 Difficult Interpersonal Experiences

##### 2.4.2.1 Interpersonal Stress

The Adolescent Stress Questionnaire (ASQ; Byrne et al. 2007) was used to assess adolescents' perceptions of stressful events in three domains: *family‐related stress* (4 items), *peer‐related stress* (4 items), and *romantic relationship stress* (3 items). Cronbach alphas indicated good reliability for stress related to families (*α*_*t*1_ = 0.84, *α*_*t*2_ = 0.86, *α*_*t*3_ = 0.88), peers (*α*_*t*1_ = 0.82, *α*_*t*2_ = 0.78, *α*_*t*3_ = 0.81), and romantic relationships (*α*_*t*1_ = 0.79, *α*_*t*2_ = 0.82, *α*_*t*3_ = 0.87).

##### 2.4.2.2 Peer Victimization

We used the Short‐Personal Experiences Checklist (PECK; Hunt et al. 2012) to measure peer victimization by *relational* and *physical aggression*. Participants reported on the number of times in the month before assessment that they had been relationally victimized (6 items, e.g., “Other kids say mean things behind my back”) and physical victimized (4 items. e.g., “Other kids hit me”). Items were rated on a scale ranging from *never* (1) to *daily* (5). For reliability, *α*_*t*1_ = 0.83, *α*_*t*2_ = 0.86, *α*_*t*3_ = 0.88 for relational; *α*_*t*1_ = 0.84, *α*_*t*2_ = 0.88, *α*_*t*3_ = 0.91 for physical victimization.

#### 2.4.3 Intrapersonal Issues

##### 2.4.3.1 Internalizing Symptoms

We assessed symptoms of depression and anxiety. For depressive symptoms, we used the 16‐item Center for Epidemiological Studies‐Depression Scale (CES‐D; Radloff 1977), revised for use with adolescents and translated into Swedish (CES‐DC, Swedish; Olsson and von Knorring 1997). Responses were given on a Likert‐like scale ranging from 0 (*not at all*) to 3 (*a lot)*. Cronbach's alphas were: *α*_*t*1_ = 0.95, *α*_*t*2_ = 0.90, *α*_*t*3_ = 0.91.

For anxiety, we used the OASIS scale (Overall Anxiety Severity and Impairment; Norman et al. 2006) to assess symptoms of anxiety. The participants responded to five questions about the frequency and intensity of symptoms of anxiety, and interference with daily behaviors and social interactions. Cronbach's alphas were all 0.88 for waves 1 to 3.

##### 2.4.3.2 Emotion‐Regulation Issues

Adolescents' tendencies to ruminate were measured using the 10‐item Children's Response Styles Scale (CRSS; Rood et al. 2010) (e.g., “When I'm feeling sad, I think about the things that have happened over and over again”). Possible responses ranged from *never* (1) to *all the time* (4), and alphas indicated good reliability (*α*_*t*1_ = 0.93, *α*_*t*2_ = 0.92, *α*_*t*3_ = 0.94).

To assess adolescents' tendencies to worry excessively, we used the Penn State Worry Questionnaire for Children (PSWQ‐C; Chorpita et al. 1997). Participants responded to 13 items (e.g., “When I'm under pressure, I worry a lot.”), potentially ranging from *not at all true* (1) to *always true* (4). The scale showed good reliability (*α*_*t*1_ = 0.82, *α*_*t*2_ = 0.85, *α*_*t*3_ = 0.85).

Anger dysregulation in the methods of dysregulated outward expressions of anger and low anger awareness were assessed using items described by Tilton‐Weaver et al. (2019). Participants were asked “What happens when you get REALLY ANGRY with someone?” They indicated their agreement (*I don't agree at all* = 1 to *I agree completely* = 4) with 5 items for each scale (e.g., “I behave aggressively, even though I don't want to” for dysregulated expressions of anger and “I try to understand what made me angry” (reverse coded) for low anger awareness. These scales had good internal consistency across all waves (dysregulated expressions: *α*_*t*1_ = 0.79, *α*_*t*2_ = 0.77, *α*_*t*3_ = 0.77; awareness: *α*_*t*1_ = 0.79, *α*_*t*2_ = 0.75, *α*_*t*3_ = 0.78).

#### 2.4.4 Factor Analysis

To reduce data, we factor analyzed scales within each waves modeling stress scales for *stress*, physical and relational victimization for *peer victimization*, symptoms of depression and anxiety for *internalizing symptoms*, rumination and worry for *repetitive negative thinking*, and dysregulated expressions of anger and low awareness as *anger dysregulation*. At each wave, the fit indices indicated good fits to the data (more information is available upon request). Factor scores were used in subsequent analyses.

### 2.5 Plan of Analysis

Using MPlus (v8.10; Muthén and Muthén 2017) and following procedures outlined by Nylund‐Gibson et al. (2014) and Masyn (2013), we modeled latent two‐class to five‐class solutions, separately for each wave, including only participants who had reported NSSI at that wave. At each wave, the latent profile model and the number of classes that best represented the data were selected based on six criteria (Muthén and Muthén 2017): (1) lowest replicated log‐likelihood, (2) lowest Akaike Information Criteria (AIC; Akaike 1973), and sample‐size adjusted Bayesian Information Criteria (saBIC; Sclove 1987), (3) highest entropy, (4) average posterior probabilities > 0.75 (AvPP; Muthén and Muthén 2017), (5) Lo‐Mendell‐Rubin Adjusted Likelihood Ratio Test (LMR‐LRT; Lo 2001), where nonsignificance indicates that the added profile has not contributed to recovering heterogeneity; and (6) the meaning of the best‐fitting models. After identifying the best‐fitting models, we used BCH procedures to examine cross‐sectional differences across subgroups.

## 3 Results

### 3.1 Descriptive Statistics

The frequencies of each method are reported in Supporting Information: Table 1 (online). At all three time points, preventing wounds from healing was the most commonly endorsed method of NSSI, along with banging the head, cutting, and scratching with sharp objects. Less common methods included burning, biting, and sticking sharp objects into the skin. Many reported using more than one method (reporting 2–9 methods: 50% at T1, 53% at T2, and 57% at T3).

### 3.2 Identification of Latent Classes

We concluded that the three‐class solutions from the invariant sigma‐diagonal models fit the data best at all three waves (see Table 1). Although 5% is a typical cut‐off point (Nylund‐Gibson and Choi 2018), we considered slightly smaller classes reasonable when the LMR‐LRT indicated that its inclusion added significantly to recovered heterogeneity.

**Table 1:** Results of latent profile analyses for T1, T2, and T3.

Model	LL	AIC	saBIC	En‐tropy	AvPP	% small	LMR‐aLRT	*p* value
T1								
Inv., Σ diagonal								
2 class	−4290.50	8639.01	8658.56	0.991	0.993	11	1028.27	0.03
**3 class**	**−4027.58**	**8135.15**	**8162.12**	**0.997**	**0.997**	**4**	**517.81**	**0.35**
4 class^b^	−3905.32	7912.63	7947.01	0.998	0.999	1	240.78	0.52
5 class^ab^	−3815.61	7755.22	7797.02	0.994	0.997	1	176.89	0.81
Inv., Σ nondiagonal								
2 class	−3998.69	8127.38	8171.20	1.000	1.000	5	401.92	0.21
3 class	−3854.91	7861.82	7913.05	0.995	0.997	4	283.16	0.25
4 class^a^	−3752.10	7678.20	7736.85	0.995	0.998	1	202.47	0.66
5 class^a^	−3680.11	7556.22	7622.29	0.994	0.996	3	145.34	0.81
Var., Σ nondiagonal								
2 class^a^	−4010.39	8240.77	8314.93	0.999	0.999	3	383.60	0.55
3 class^abc^	−4202.77	8665.55	8753.19	—	—	—	—	—
4 class^abc^	−3912.33	8268.67	8418.33	—	—	—	—	—
5 class^abc^	−3816.46	8188.92	8376.34	—	—	—	—	—
T2								
Inv., Σ diagonal								
2 class	−4897.65	9853.29	9873.42	0.984	0.990	14	826.56	0.01
**3 class**	**−4691.60**	**9463.20**	**9490.96**	**0.988**	**0.986**	**5**	**405.80**	**0.30**
4 class	−4572.41	9246.82	9282.22	0.986	0.989	3	234.74	0.64
5 class^bc^	−4419.62	8963.25	9006.28	0.992	0.998	1	305.57	0.00
Inv., Σ non‐diagonal								
2 class	−4690.17	9510.33	9555.45	0.999	0.999	6	344.09	0.04
3 class	−4542.32	9236.63	9289.38	1.000	1.000	1	293.06	0.21
4 class	−4427.50	9028.99	9089.38	0.997	0.997	3	251.26	0.33
5 class^a^	−4328.06	8852.11	8920.13	0.997	0.997	2	195.85	0.56
Var., Σ diagonal								
2 class^a^	−7263.71	34,789.43	34,880.3	—	—	—	0.00	0.50
Var., Σ nondiagonal								
2 class^a^	−4633.20	9486.41	9562.7	1.000	1.000	5	461.94	0.66
3 class^b^	−4864.88	10,061.76	10,176.97	—	—	—	0.00	0.50
4 class^bc^	−4864.88	10,173.76	10327.84	—	—	—	—	—
5 class^bc^	−4576.98	9709.96	9902.91	—	—	—	—	—
T3								
Inv., Σ diagonal								
2 class	−4647.28	9352.56	9369.49	0.990	0.998	14	1094.47	0.02
**3 class**	**−4425.23**	**8930.46**	**8953.82**	**0.990**	**0.994**	**6**	**437.20**	**0.15**
4 class	−4309.66	8721.32	8751.10	0.990	0.989	4	227.55	0.63
5 class	−4216.05	8556.10	8592.31	0.991	0.990	4	184.31	0.26
Inv., Σ non‐diagonal								
2 class	−4381.78	8893.56	8931.52	0.997	0.995	7	416.65	0.01
3 class^a^	−4201.47	8554.93	8599.32	0.997	0.993	4	355.02	0.05
Var., Σ non‐diagonal								
2 class	−4228.90	8677.80	8742.04	0.995	0.997	14	726.74	0.49
3 class^ab^	−4593.39	9518.79	9615.73	—	—	—	0.00	50
4 class^abc^	−4362.30	9168.60	9298.24	—	—	—	—	—
5 class^abc^	−4333.89	9151.78	9293.11	—	—	—	—	—

*Note:* Overall *n* = 630; T1 = 346, T2 = 353, T3 = 316. Inv. = invariant; Var. = varying. Bolded models were selected as the best fitting models and were used for the latent transition analyses. All 2–5 class models were tested; but only models that terminated normally were included in the table.

Abbreviations: AIC, Akaike Information Criteria; AvPP, average posterior probabilities; LMR‐aLRT, Lo–Mendell–Rubin adjusted likelihood ratio test; saBIC, sample‐size adjusted Bayesian Information Criteria.

^a^ Log‐likelihood did not replicate, indicating local maxima.

^b^ Although models converged, other indications of model deficiencies were noted (empty cells in the joint distribution, non‐positive definite matrices).

^c^ Draws for LMR‐aLRT did not converge.

**Figure 1:**
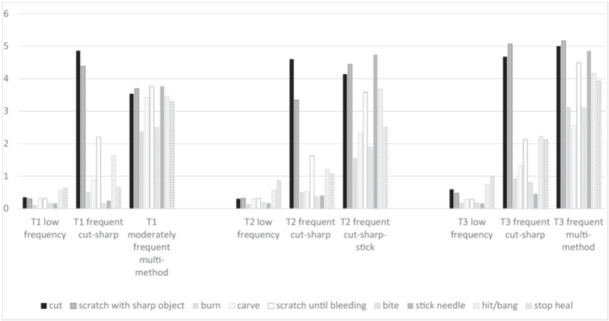
Estimated indicator means for latent classes at T1, T2, and T3.

The latent profiles for each wave are depicted in Figure 1; counts and indicator means are presented in Table 2. At all three time points, the profile including the greatest percentage of participants consisted of adolescents reporting low frequencies across NSSI methods (T1: 87%, *n* = 301; T2: 80%, *n* = 283; T3: 84%, *n* = 265). We gave this subgroup the label of *low frequency*. A second, smaller subgroup emerged with profiles indicating higher frequencies of cutting and scraping skin with a sharp object in all waves (T1: 9%, *n* = 32; T2: 15%, *n* = 54; and T3: 10%, *n* = 33). We labeled the subgroup with this profile as *frequent cutting and scraping with sharp objects*. Finally, across all waves, very small subgroups of adolescents with profiles of moderate or high frequencies combined with high levels of versatility were evident (T1: 4%, *n* = 13; T2: 5%, *n* = 16; and T3: 6%, *n* = 18). We labeled these subgroups *moderately frequent multi‐method* at T1, *frequent cutting, scraping, and sticking skin with sharp objects* at T2, and *frequent multi‐method* at T3.

**Table 2:** Estimated profile means and standard errors for each self‐injury method, with final classification counts.

	T1						T2						T3					
	Low frequency		Frequent cutting and scraping skin with sharp objects		Moderately frequent multi‐method		Low frequency		Frequent cutting and scraping skin with sharp objects		Frequent cutting, scraping, and sticking skin with sharp objects		Low frequency		Frequent cutting and scraping skin with sharp objects		Frequent multi‐method	
Method of self‐injury	*M*	*SE*	*M*	*SE*	*M*	*SE*	*M*	*SE*	*M*	*SE*	*M*	*SE*	*M*	*SE*	*M*	*SE*	*M*	*SE*
Cutting wrists, arms, other body parts	0.35	0.04	4.86	0.24	3.54	0.51	0.31	0.04	4.61	0.21	4.14	0.58	0.60	0.08	4.68	0.38	5.00	0.38
Scratching with sharp object	0.31	0.04	4.39	0.30	3.69	0.47	0.32	0.04	3.35	0.29	4.44	0.54	0.48	0.06	5.07	0.26	5.16	0.31
Burning with cigarette, lighter, matches	0.12	0.03	0.51	0.22	2.39	0.48	0.14	0.04	0.52	0.17	1.56	0.54	0.19	0.05	0.93	0.29	3.13	0.70
Carving	0.34	0.05	0.88	0.25	3.42	0.57	0.31	0.05	0.53	0.13	2.32	0.68	0.39	0.04	1.32	0.33	2.57	0.67
Scratching until wound or bleeding	0.31	0.04	2.21	0.45	3.77	0.49	0.32	0.05	1.62	0.30	3.59	0.65	0.39	0.05	2.13	0.44	4.50	0.33
Biting	0.17	0.03	0.16	0.11	2.50	0.48	0.20	0.04	0.37	0.12	1.89	0.55	0.19	0.04	0.81	0.27	3.11	0.59
Sticking sharp objects into skin	0.17	0.03	0.25	0.10	3.77	0.35	0.17	0.03	0.41	0.15	4.74	0.42	0.16	0.03	0.45	0.17	4.85	0.38
Hitting or banging head	0.58	0.06	1.64	0.38	3.46	0.51	0.56	0.07	1.22	0.24	3.70	0.65	0.74	0.08	2.23	0.44	4.16	0.46
Preventing wounds from healing	0.65	0.06	0.66	0.20	3.31	0.54	0.87	0.09	1.07	0.24	2.52	0.71	0.98	0.10	2.14	0.46	3.93	0.62
Final class count (%)	*n* = 301 (87%)		*n* = 32 (9%)		*n* = 13 (4%)		*n* = 283 (80%)		*n* = 54 (15%)		*n* = 16 (5%)		*n* = 265 (84%)		*n* = 33 (10%)		*n* = 18 (6%)	

*Note:* Overall *n* = 630; T1 = 346, T2 = 353, T3 = 316 (cases with no NSSI not included). All items of methods of self‐injury ranged from 0 (*never*) to to 6 (*6 or more times*).

Abbreviations: NSSI, Nonsuicidal self‐injury; SE, sandard error.

### 3.3 Analysis of Profile Subgroup Differences

According to BCH comparisons, significant differences were evident across all waves (see Table 3 for these estimates). First, the subgroups differed in severity: At T1 and T3, adolescents in the *low frequency* and *frequent cutting and scraping with sharp objects* profiles reported less need for medical attention than those in the *moderately frequent multi‐method* profile. A similar pattern emerged at T2, but the *frequent cutting and scraping with sharp objects* profile did not significantly differ from the *frequent cutting and scraping with sharp objects* profile.

**Table 3:** BCH comparisons of latent profiles, T1–T3.

			Latent profile, T1						
	Low frequency		Frequent cutting and scraping skin with sharp objects			Moderately frequent multi‐method		Omnibus	
Correlate	*M*	*SE*	*M*	*SE*		*M*	*SE*	*χ* ^2^	*p*‐value
Self‐injury severity	0.08^a^	0.03	0.32^a^	0.20		1.92^b^	0.49	15.33	< 0.001
Gender	Girls^a^ (54.2%)	—	Girls^b^ (87.5%)	—		Girls^a^ (53.8%)		26.44	< 0.01
Difficult interpersonal experiences									
Stress	0.12^a^	0.06	1.12^b^	0.18		1.02^b^	0.43	31.46	< 0.001
Peer victimization	0.11^a^	0.06	0.83^b^	0.17		1.50^b^	0.54	21.92	< 0.001
Intrapersonal issues									
Internalizing symptoms	0.13^a^	0.06	1.54^b^	0.20		1.09^b^	0.43	52.26	< 0.001
Repetitive negative thinking	0.15^a^	0.06	1.29^b^	0.15		0.35^a^	0.38	49.13	< 0.001
Anger dysregulation	0.15^a^	0.06	0.32^a^	0.16	—	0.39^a^	0.31	4.33	0.12

	Latent profile, T2								
	Low frequency		Frequent cutting and scraping skin with sharp objects			Frequent cutting and scraping or sticking skin with sharp objects		Omnibus	
Correlate	*M*	*SE*	*M*	*SE*		*M*	*SE*	*χ* ^2^	*p*‐value
Self‐injury severity	0.09^a^	0.03	0.38^ab^	0.17		1.19^b^	0.43	9.28	0.01
Gender	Girls^a^ (56.2%)	—	Girls^b^ (87.0%)	—		Girls^b^ (81.3%)		34.68	< 0.001
Difficult interpersonal experiences									
Stress	0.07^a^	0.06	0.76^b^	0.16		1.06^b^	0.33	24.81	< 0.001
Peer victimization	0.04^a^	0.06	0.06^a^	0.11		0.59^a^	0.33	2.66	0.27
Intrapersonal issues									
Internalizing symptoms	0.06^a^	0.06	0.98^b^	0.14		1.58^c^	0.18	91.47	< 0.001
Repetitive negative thinking	0.07^a^	0.06	0.75^b^	0.13		1.14^b^	0.16	58.48	< 0.001
Anger dysregulation	0.12^a^	0.06	0.33^a^	0.13		0.39^a^	0.19	3.53	0.17

	Latent profile, T3								
	Low frequency		Frequent cutting and scraping skin with sharp objects			Frequent multi‐method		Omnibus	
Correlate	*M*	*SE*	*M*	*SE*		*M*	*SE*	*χ* ^2^	*p*‐value
Self‐injury severity	0.09^a^	0.03	0.46^a^	0.19		1.56^b^	0.51	11.87	0.003
Gender	Girls^a^ (58.9%)	—	Girls^b^ (90.9%)	—		Girls^ab^ (72.2%)		30.23	< 0.001
Difficult interpersonal experiences									
Stress	0.12^a^	0.06	1.08^b^	0.18		0.68^ab^	0.32	26.81	< 0.001
Peer victimization	0.05^a^	0.07	0.25^ab^	0.12		1.11^b^	0.44	7.47	0.02
Intrapersonal issues									
Internalizing symptoms	0.15^a^	0.06	1.32^b^	0.18		1.58^b^	0.23	69.44	< 0.001
Repetitive negative thinking	0.11^a^	0.06	0.83^b^	0.16		0.69^b^	0.25	20.90	< 0.001
Anger dysregulation	0.09^a^	0.06	0.51^b^	0.16		0.90^b^	0.21	18.94	< 0.001

*Note:* Profiles sharing superscripts do not differ significantly.

Abbreviation: SE, standard error.

Across all waves, every profile consisted of more girls than boys, but at T1 and T2, the *low frequency* profiles consisted of fewer girls than the other profiles. At T3, only the *low frequency* and *frequent cutting and scraping with sharp objects* profiles differed. This is likely due to the *low frequency* profile having more boys at this wave than earlier (58.9% at T1 compared to 54.2% at T2 and 56.2% at T3). Notably, the number of girls in the *frequent cutting and scraping with sharp objects* profiles supported our expectation that these methods of NSSI would be reported more by girls than boys.

Concerning interpersonal experiences, stressful experiences had nearly the same pattern across all three waves: adolescents with the *low frequency* profile reported less stress than adolescents in other profiles. At T3 only, the *low frequency* profile differed only from the *frequent cutting and scraping with sharp objects* profile. Victimization by peers significantly differentiated between profiles at T1 and T3. Similar to the pattern for stress, at T1 adolescents with the *low frequency* profile reported significantly less victimization than adolescents with other profiles. At T3, the *low frequency* profile differed only from the *frequent multi‐method* profile, reporting less victimization.

Regarding intrapersonal issues, at T1 and T3, the low profile reported fewer internalizing symptoms than the other profiles. At T2, all three differed: the *low frequency* reported the fewest symptoms, *frequent cutting and scraping with sharp objects* in the middle, and *frequent cutting and scraping with sharp objects* with the highest. For repetitive negative thinking, only the *frequent cutting and scraping with sharp objects* profile differed from the others, with higher levels than either those with *low frequency* or with *moderately frequent multi‐method* profiles. At T2 and T3, the *low frequency* differed from the other profiles, reporting the least repetitive negative thinking. Finally, differences in anger dysregulation were only apparent at T3, with the same pattern just described: compared to others, adolescents with the *low frequency* profile reported the least dysregulation.

### 3.4 Profile Change

Due to having profiles that were rather small, we were unable to use *MPlus* to model latent profile transitions. Instead, we saved the most likely classification for each case and conducted *χ* ^2^ analyses, examining change from T1 to T2, T1 to T3, and T2 to T3. For these analyses, we included individuals who reported no NSSI at one of the waves, assigning them to a “No NSSI” subgroup.

Significant change was found in profile membership (see Table 4), from T1 to T2 (*χ* ^2^[9] = 154.26, *p* < 0.001), from T1 to T3 (*χ* ^2^[9] = 72.67, *p* < 0.001) and from T2 to T3 (*χ* ^2^[9] = 148.39, *p* < 0.001). Overall, the patterns of change were relatively similar.

**Table 4:** Profile change, based on most‐likely classifications.

	Cell *n* and percentage of earlier profiles			
	T2 profiles			
T1 profiles	None (*n* = 277)	Low frequency (*n* = 283)	Frequent cutting and scraping skin with sharp objects (*n* = 54)	Frequent cutting and scraping or sticking skin with sharp objects (*n* = 16)
None (*n* = 284)	106 (37%)	163 (57%)	12 (4%)	3 (1%)
Low frequency (*n* = 301)	162 (54%)	109 (36%)	25 (8%)	5 (2%)
Frequent cutting and scraping skin with sharp objects (*n* = 32)	3 (9%)	8 (25%)	16 (50%)	5 (16%)
Moderately frequent multi‐method (*n* = 13)	6 (46%)	3 (23%)	1 (8%)	3 (23%)
*χ* ^2^(9) = 154.26, *p* < 0.001				

	T3 profiles			
T1 profiles	None (*n* = 314)	Low frequency (*n* = 265)	Frequent cutting and scraping skin with sharp objects (*n* = 33)	Frequent multi‐method (*n* = 18)
None (*n* = 284)	114 (40%)	150 (53%)	13 (5%)	7 (2%)
Low frequency (*n* = 301)	185 (61%)	97 (32%)	12 (4%)	7 (2%)
Frequent cutting and scraping skin with sharp objects (*n* = 32)	6 (18%)	16 (50%)	8 (25%)	2 (6%)
Moderately frequent multi‐method (*n* = 13)	9 (69%)	2 (15%)	0 (0%)	2 (15%)
*χ* ^2^(9) = 72.67, *p* < 0.001				

	T3 profiles			
T2 profiles	None (*n* = 314)	Low frequency (*n* = 265)	Frequent cutting and scraping skin with sharp objects (*n* = 33)	Frequent multi‐method (*n* = 18)
None (*n* = 277)	145 (52%)	119 (41%)	8 (3%)	5 (2%)
Low frequency (*n* = 283)	159 (56%)	115 (41%)	8 (3%)	1 (0.4%)
Frequent cutting and scraping skin with sharp objects (*n* = 54)	8 (15%)	25 (46%)	14 (26%)	7 (13%)
Frequent cutting and scraping or sticking skin with sharp objects (*n* = 16)	2 (13%)	6 (38%)	3 (19%)	5 (31%)
*χ* ^2^(9) = 148.39, *p* < 0.001				

From T1 to T2, the greatest change was between those who reported no NSSI and the *low frequency* subgroups, who tended to switch places. However, these two groups were more stable than the most frequent and versatile subgroups (37% and 36%, respectively, compared to 23% being classified first as *moderately frequent multi‐method* and then into the *frequent cutting and scraping or sticking skin with sharp objects*). The most stable grouping from T1 to T2 were those classified in the *frequent cutting and scraping skin with sharp objects*, with 50% being classified in these similar profiles over time.

These same groupings were less stable over 2 years (i.e., T1–T3), as might be expected, with 40%, 32%, and 25% classified in the same subgroups (i.e., of no NSSI, *low frequency*, *and frequent cutting and scraping skin with sharp objects*, respectively). By contrast, only 15% remained in the subgroups with the highest frequency and versatility.

From T2 to T3, the subgroups were notably more stable, with 52%, 40%, and 26% classified in the same subgroups of no NSSI, *low frequency*, *and frequent cutting and scraping skin with sharp objects*, respectively. The subgroups with the highest levels of frequency and versatility were also more stable than in the previous lag, with 31% classified in the *frequent cutting and scraping or sticking skin with sharp objects* subgroup at T2 and the *frequent multi‐method* subgroup at T3.

### 3.5 Post Hoc Comparisons of Profile Change

As transitional analysis was not possible, we were only able to explore what predicted change in subgroup membership. To preserve power, we chose to compare those who maintained a subgroup (“stayers”) to those who moved (“movers”) within each subgroup. For example, we compared those who were classified at T1 and T2 as *low frequency* (i.e., *low frequency stayers*) to adolescents who were initially classified as *low frequency* and moved to another subgroup at T2. For the multiform profiles, we treated individuals who maintained a multiform profile across time “stayers” (i.e., T1 *moderately frequent multi‐method* and T2 *frequent cutting and scraping or sticking skin with sharp objects;* T2 *frequent cutting and scraping or sticking skin with sharp objects* and T3 *high frequency multiform*). Using one‐way ANOVAs (with Games‐Howell post‐hoc comparisons) and chi‐square, we tested whether gender, age, immigrant/nonimmigrant background, and the interpersonal and intrapersonal variables predicted change in subgroupings. To adjust for pairwise inflation of Type I error, we adjusted the alpha level for the *F* test to 0.005.

In these exploratory analyses, we found change predicted for only one group at T1: those who started in the *low frequency* subgroup. Those who were stayed T1–T2 reported significantly less interpersonal stress (*F* = 9.84, *p* < 0.001; *M*_diff_ = 0.41, *SE* = 0.12, *p* = 0.004), less internalizing (*F* = 11.14, *p* < 0.001; *M*_diff_ = 0.43, *SE* = 0.11, *p* < 0.001), less repetitive negative thinking (*F* = 7.79 *p* = 0.002; *M*_diff_ = 0.40, *SE* = 0.11, *p* = 0.002), and less dysregulated anger (*F* = 5.09, *p* = 0.002; *M*_diff_ = 0.39, *SE* = 0.12, *p* = 0.008) than adolescents who reported *no NSSI* at T2. In addition, comparatively more boys (64.5%) than girls (44.8%) moved to the *no NSSI* subgroup, whereas gender was more equally split among those who stayed in the *low frequency* subgroup (42.2% girls, 57.8% boys; *χ* ^2^ = 21.75, *p* < 0.001). No other significant differences were found.

## 4 Discussion

Our aim was to explore heterogeneity in the methods of NSSI in early to middle adolescence. Our results revealed three rather similar profiles at each wave: consistently large and low frequency profiles, consistently smaller profiles with elevated frequencies of cutting and scraping skin, and small profiles reporting multiple methods of NSSI, from moderate at T1 to increasingly higher frequencies at T2 and T3. The *low frequency* profiles replicate previous research, such as Somer et al.'s (2015) low class and Klonsky and Olino's (2008) mild NSSI class. Unlike ours, their classes were much smaller (29% and 17%, respectively). This difference could be due to our emphasis on early to middle adolescence, instead of high school (Somer at al. 2015) or college/university students (Hamza and Willoughby 2014; Klonsky and Olino 2008). These variations may mean that preferences for one or two methods increases late in adolescence after earlier experimentation.

A few issues are worth noting about profile differences. First, our results suggest that a priori categorizations of severity, based on the assumed degree of skin damage, may need revising. In our results, adolescents who reported higher levels of cutting and scraping—NSSI that damages skin—did not significantly differ from those with low levels across all methods, in terms of needing medical attention. Although the lack of differences may be due to small cell sizes, this warrants further attention. We recommend assessing severity separately for each method (Lloyd‐Richardson et al. 2007).

As we expected, girls were over‐represented in the sample of those reporting at least one incident of NSSI. Compared to the *low frequency*, the percentage of girls in other profiles was even higher. As the literature on gender has been somewhat mixed, our results provide additional evidence that girls engage in more frequent NSSI using multiple methods than do boys.

Generally, adolescents classified with the *low frequency* profile evidenced the fewest interpersonal and intrapersonal difficulties. This was the case for stressful experiences (T1 and T2), peer victimization (T1 only), internalizing symptoms, repetitive negative thinking (T2 and T3), and anger dysregulation (T3). This pattern of findings is somewhat consistent with “experimental” profiles.

In many ways, the other two profiles did not differ from each other. However, at T2 all three profiles varied in stressful experiences, where such experiences seemed to increase with frequency and versatility of NSSI. The pattern changed at T3, with the higher use of cutting and scraping with sharp objects profile reporting the most stressful experiences, relative to the others. The reason for this pattern shift is unclear. Perhaps stressful experiences concentrate in one context for those classified as *frequent multi‐method* and drop in other contexts.

Peer victimization patterns also differed across waves. At T1, adolescents with *low frequency* profiles reported the least victimization. At T2, the profiles did not differ significantly. At T3, only the *low frequency* and *frequent multi‐method* profiles differed. In other words, victimization did not follow a simple pattern but appeared to be consistently high in profiles marked by versatility. The pattern also shifted for repetitive negative thinking, but in a way that suggested repetitive negative thinking is tied to frequency of NSSI, as opposed to versatility. That is, the profiles with the highest levels of repetitive negative thinking were consistently those reporting more frequent NSSI. Frequency and versatility of methods appear to be important for understanding potential markers of NSSI.

It is also notable that across smaller profiles, NSSI using sharp objects is more frequent. Why does using sharp objects look different from other methods? Perhaps these methods are viewed as more immediately effective in terms of the functions they serve (Bachtelle and Pepper 2015) or the level of pain desired (Brausch and Muehlenkamp 2014). Alternatively, media images may influence what methods adolescents use, with “cutting” or using sharp objects (and the pejorative label of “cutter”) being the method many people associate with NSSI.

Finally, we noted a great deal of change in profile membership over time. Instability was the rule, rather than the exception, as even the most stable subgroupings had only 52% classified in similar profiles over time. This may reflect real instability in the frequency and versatility of methods used, an artifact of measurement and analyses (e.g., our inclusion of only those who reported engaging in NSSI in the wave analyzed), or that many participants received treatment. In addition, we found few differences between those who maintained a similar profile over time (stayers) and those who moved. The only differences found were between T1 and T2, for the stayers in *low frequency* profiles compared to those later reported no NSSI. The lack of other differences could be due to low power or similar issues to what we just described. As our study is one of the first tracking profiles over time, further research, including determining if treatment has occurred, is warranted.

### 4.1 Strengths, Limitations, and Future Directions

Our study had some important strengths, including a relatively large community‐based sample of adolescence during the time in which NSSI tends to emerge and rise. In addition, we used a measure of NSSI that tapped multiple methods, rather than a single item. Although the use of a single item may provide more consistent reports of NSSI than assessing separate methods (Robinson et al. 2021), our measure allowed us to examine the frequency of each method and versatility in use. Notably, researchers comparing single‐item and multi‐item inventories of NSSI has interpreted such variations as indicating that individuals do not always identify their behaviors as self‐injury. The measure we used asked participants to report intentional harm to self (without suicidal intent). It is possible that such measures underestimate some methods of self‐injury (e.g., cutting).

Despite the unique contributions of this study, the results need to be interpreted in light of its limitations. First, we chose analytic strategies, out of necessity, that may have affected the results. Limiting analyses to adolescents who reported NSSI within a specific wave precluded BCH comparison to adolescents who had not engaged in NSSI. Although using the classifications to examine transitions and explore differences ignores uncertainty in classification, and asserting “no NSSI” for those who reported none may also introduce measurement error, analytical choices were limited. Nonetheless, our choices may have led to instability in classification over time, with significant change due to selection rather than change in NSSI behaviors. We thus urge caution in interpreting our results, but suggest that future research continue to explore the patterns of methods, with the hope that analytical strategies are developed that overcome these limitations.

Second, NSSI can vary daily (Hamza and Willoughby 2015). Using daily diaries would reveal more intensive changes and reduce the likelihood of recall errors (Bresin 2014). In addition, we used only self‐reports, which may reflect bias, although estimates of common variance are below those typically viewed as common method variance (~42%, lower than the 50% cutoff). Given the levels in our sample, though, under‐reporting was not evident.

Another drawback was that the sample was predominantly native‐born Swedes, eliminating ethnic‐based comparisons. We note that unlike other samples, where cutting is most commonly reported, our sample most commonly reported preventing wounds from healing. With an increasing number of adolescents reporting engagement in NSSI (Zetterqvist et al. 2013), we can only speculate that this may be a cultural variation that has not yet been documented. As cultural‐specific processes are possible, we recommend replication in other samples.

Finally, one class was quite small, considering suggested cut‐offs for more normative issues (Jung and Wickrama 2008). This could be expected in community samples, where most are not involved in NSSI. However, entropy and posterior probabilities indicated very little misclassification. Because of these smaller profiles, we were also unable to conduct transition analyses and comprehensively examine what predicted change. Again, caution in interpretation is warranted, particularly when comparing differences over time, as the small size likely contributes to instability in classification as increasing error of estimation. Combined, these issues lead us to recommend replication in larger, more diverse community samples.

## 5 Conclusions

Our study adds to the developmental record, a glimpse into the methods of NSSI during early and middle adolescence. The results are suggestive of developmental patterns, where low and elevated levels of cutting and scratching are consistent across this period, but other configurations move toward higher frequencies across multiple methods. The evidence from this study can guide further research efforts, with the aim of identifying signs that help practitioners know who to target for treatment.

## Ethics Statement

This study, including the data collection, was conducted in compliance with the 1964 Declaration of Helskinki, ethics guidelines from the Swedish Research Council (Vetenskapsrådet), and in full compliance with the EU and Sweden's laws protecting personal data (e.g., GDPR).

## Consent

Before data collection, ethics approval was sought and granted by the Regional Ethics Board (Uppsala, dnr: 2013/384), including approval to use passive parental consent and active adolescent assent.

## Conflicts of Interest

The authors declare no conflicts of interest.

## Supporting information

JAD 2025‐0080‐R1 NSSI‐LPA‐METHODS SuppTable 250429.docx.

## Data Availability

In compliance with Swedish laws governing protection of highly personal information, the data set generated for this study are not currently available to the public. Restricted access may be granted upon reasonable request and with permission of authorities at Örebro University.
