# Supplementary material for: Examining the Methods Adolescents Use in Nonsuicidal Self‐Injury: A Multi‐Wave Latent Profile Analysis
Source: J Adolesc. 2025 May 28;97(6):1530–46. doi: 10.1002/jad.12516 (PMC12318471; doi:10.1002/jad.12516)
Supplement: Supplementary file 1 — JAD 2025‐0080‐R1 NSSI‐LPA‐METHODS SuppTable 250429.docx. [file JAD-97-1530-s001.docx]

Supplemental Table 1

*Demographic Characterstics For the Full Sample (n = 630) and Sub-Samples*

| Demographic variable | Full sample  (T1 values) | T1  sample | T2  sample | T3  sample |
| --- | --- | --- | --- | --- |
| *N* | 630 | 346 | 353 | 316 |
| Adolescent gender | 56.0% girls | 57.0% girls | 62.0% girls | 63.0% girls |
| Adolescent age | *M* = 13.59  *SD* = .67 | *M* = 13.61  *SD* = .65 | *M* = 14.75  *SD* = .70 | *M* = 15.69  *SD* = .73 |
| Adolescents’ birthplace |  |  |  |  |
| Sweden | 89.4% | 88.6% | 90.6% | 91.3% |
| Outside of Sweden | 10.6% | 11.4% | 9.4% | 8.7% |
| Mothers’ birthplace |  |  |  |  |
| Sweden | 77.1% | 75.0% | 79.0% | 81.7% |
| Another Scandinavian country | 2.3% | 2.1% | 2.0% | 2.6% |
| Another European country | 6.3% | 6.8% | 3.7% | 5.1% |
| Outside of Europe | 14.3 | 16.2% | 14.7% | 10.6% |
| Fathers’ birthplace |  |  |  |  |
| Sweden | 78.2 | 74.6% | 77.1% | 82.6% |
| Another Scandinavian country | 1.8 | 1.7% | 1.1% | 3.2% |
| Another European country | 5.9 | 7.3% | 5.9% | 4.5% |
| Outside of Europe | 14.1 | 15.5% | 14.2% | 9.7% |
| Parents divorce / separation |  |  |  |  |
| Divorced or separate | 35.1 | 35.0% | 37.1% | 37.0% |
| Not divorced/separated | 64.9 | 65.0% | 60.6% | 60.4% |
| Adults they live with |  |  |  |  |
| Both mother and father | 69.1 | 69.8% | 65.4% | 65.8% |
| Sometimes mother, sometimes father | 15.9 | 14.7% | 12.9% | 12.0% |
| With father | 3.4 | 2.9% | 3.7% | 4.1% |
| With mother | 10.5 | 11.7% | 15.4% | 13.9% |
| Someone else | 1.1 | 0.9% | 2.6% | 2.5% |
| Family owns a car or van |  |  |  |  |
| None | 5.6 | 5.3% | 5.7% | 2.9% |
| One | 41.9 | 41.8% | 39.7% | 37.6% |
| Two or more | 52.5 | 52.9% | 5.6% | 59.5% |
| Adolescent has own bedroom |  |  |  |  |
| Yes | 90.1 | 89.3% | 92.9% | 93.9% |
| No | 9.9 | 10.7% | 7.1% | 6.1% |
| Family travel for holiday, past year |  |  |  |  |
| None | 14.1 | 15.6% | 15.5% | 17.5% |
| Once | 35.2 | 33.9% | 31.2% | 28.5% |
| Twice | 22.8 | 22.4% | 23.2% | 24.9% |
| More than twice | 27.9 | 28.0% | 30.1% | 29.1% |
| Number of computers at home |  |  |  |  |
| None | 0.5 | 0.6% | 0.3% | 0.3% |
| One | 7.1 | 7.9% | 6.0% | 6.8% |
| Two | 20.2 | 22.7% | 22.1% | 16.7% |
| More than two | 72.3 | 68.8% | 71.6% | 76.2% |
